# Is Shear-Wave Elastography a Clinical Severity Indicator of Myofascial Pain Syndrome? An Observational Study

**DOI:** 10.3390/jcm10132895

**Published:** 2021-06-29

**Authors:** Juan Antonio Valera-Calero, Sandra Sánchez-Jorge, Jorge Buffet-García, Umut Varol, Gracia María Gallego-Sendarrubias, Javier Álvarez-González

**Affiliations:** 1 Department of Physical Therapy, Universidad Camilo José Cela, Calle Castillo de Alarcón 49, Villanueva de la Cañada, 28692 Madrid, Spain; gmgallego@ucjc.edu; 2 Health Sciences Faculty, Universidad Francisco de Vitoria, Pozuelo de Alarcón, 28223 Madrid, Spain; s.sjorge.prof@ufv.es (S.S.-J.); j.buffet.prof@ufv.es (J.B.-G.); j.alvarezglez.prof@ufv.es (J.Á.-G.); 3 IE School of Human Sciences and Technology, 28006 Madrid, Spain; umut.varol@alumni.ie.edu

**Keywords:** myofascial pain syndromes, trigger points, neck pain, elasticity imaging techniques

## Abstract

Since manual palpation is a subjective procedure for identifying and differentiate Myofascial Trigger Points -MTrPs-, the use of Shear Wave Elastography -SWE- as an objective alternative is increasing. This study aimed to analyze pain pressure thresholds -PPTs- and SWE differences between active MTrPs, latent MTrPs and control points located in the upper trapezius to analyze the association of SWE features with clinical severity indicators (e.g., pain extension area, PPTs, neck pain and neck disability). An observational study was conducted to calculate the correlation and to analyze the differences of sociodemographic, clinical and SWE features on 34 asymptomatic subjects with latent MTrPs and 19 patients with neck pain and active MTrPs. Significant PPT differences between active with latent MTrPs (*p* < 0.001) and control points (*p* < 0.001) were found, but no differences between latent MTrPs and control points (*p* > 0.05). No stiffness differences were found between active MTrPs with latent MTrPs or control points (*p* > 0.05). However, significant control point stiffness differences between-samples were found (*p* < 0.05). SWE showed no significant correlation with clinical severity indicators (*p* > 0.05). No stiffness differences between active and latent MTrPs were found. Neck pain patients showed increased control point stiffness compared with asymptomatic subjects. SWE showed no association with clinical severity indicators.

## 1. Introduction

Neck pain is the fourth highest condition on number of years lived with disability with a 20% of estimated prevalence, a lifetime prevalence up to the 70% and high recurrence [1]. Current evidence suggests that a higher prevalence of Myofascial Trigger Points -MTrP- in the upper quarter muscles (e.g., upper trapezius, infraspinatus, and levator scapulae) could be found in patients with chronic neck pain compared with healthy controls [2,3].

A MTrP is defined as “a hyperirritable spot in skeletal muscle that is associated with a hypersensitive palpable nodule in a taut band. This spot is painful on manual compression and can give rise to characteristic referred pain, referred tenderness, motor dysfunction and autonomic phenomena” and could be classified as active (upon stimulation reproduce any symptom experienced by the patient, either partially or completely, whereby the symptom is recognized as a familiar experience by the patient, even though it may not be present at the moment of the examination) or latent (upon stimulation do not reproduce any symptom experienced by a subject, symptomatic or asymptomatic, and the subject does not recognize the elicited symptom as familiar) based on previous histological, neurophysiological, biochemical, sonographic and somatosensorial findings [4,5].

However, since the etiology of neck pain seems to be multifactorial [6], there is still certain controversy about whether MTrP presence is a necessary condition for Myofascial Pain Syndrome -MPS- diagnosis. Although it has been found that the prevalence and pain sensitivity of MTrPs are enough sensitive to make distinctions between patients with chronic non-traumatic neck pain and healthy subjects, the evidence supporting the high prevalence of active and latent MTrPs in patients with neck pain is based on few studies with small sample sizes and design limitations [1].

Although several diagnostic modalities aimed to understand the MTrP pathophysiology (assessed as texture-based, doppler and echo-intensity US features, microdialysis, electromyography, infrared thermography and magnetic resonance imaging), the current gold standard for the diagnosis of MTrP is the manual palpation [7]. However, since there is no consensus regarding the physical findings associated with MPS, the diagnosis accuracy is associated with the examiner clinical experience, index of suspicion, training and palpation skills [8].

Shear-wave elastography -SWE- is a non-invasive imaging technology, sensitive to tissue stiffness and develop to provide quantitative and objective data (e.g., Young modulus -kPa- and local shear wave speed -m/s-) [9] which has been used previously for assessing MTrP characteristics and showing controversial findings [10,11,12,13]. Whereas strain sonoelastography -SSE- was the first introduced elastography technique which measures physical tissue displacement parallel to the applied normal stress [14], SWE provides a more objective, reliable and valid method for quantifying muscle stiffness based on acoustically induced shear waves which travel perpendicularly to the compression waves with variate velocity depending on tissue stiffness [15].

Since current literature assessing stiffness properties of MTrPs used heterogeneous US sonoelastography methods and sample sizes are limited, the aims of this observational study were (1) to analyze pain pressure thresholds -PPTs- and SWE (e.g., Young modulus and shear-wave speed) differences between active MTrPs, latent MTrPs and control points in both samples of healthy subjects and patients with chronic neck pain and (2) to analyze the association of SWE features with MPS clinical severity indicators (e.g., pain extension area, PPTs, neck pain and neck disability).

## 2. Materials and Methods

### 2.1. Study Design

An observational study was conducted to calculate (1) the SWE and PPT differences between active MTrPs, latent MTrPs and control points and (2) the association of SWE Young modulus and shear wave speed with sociodemographic features and clinical severity indicators (e.g., pain extension area, PPTs, neck pain and neck disability). his study followed the Strengthening the Reporting of Observational studies in Epidemiology (STROBE) guidelines and checklist [16], was conducted according to the Declaration of Helsinki, and approved by the by the Institutional Ethics Committee of Clinical Research of Francisco de Vitoria University (UFV 38/2021).

### 2.2. Participants

Two consecutive samples of asymptomatic volunteers and patients with chronic neck pain were screened for eligibility criteria via flyer announcements from February 2021 to April 2021 from a private clinic located in Francisco de Vitoria University (Madrid, Spain). To be eligible for participation, volunteers had to be aged from 18 to 65 years old. Specific inclusion criteria for the asymptomatic sample were the bilateral presence of at least one latent MTrP located in the upper trapezius muscle and absence of neck pain during the previous 12 months. Specific inclusion criteria for the neck pain sample were presence of at least one active MTrP in each side of the upper trapezius muscle, neck pain evolution of at least 6 months, a Neck Disability Index (NDI) score > 8% and a Visual Analogue Scale (VAS) score > 3. Exclusion criteria were being under medical, pharmacological or physiotherapy treatment affecting pain perception or muscle tone 6 months previous to their inclusion, traumatic diseases (e.g., whiplash associated disorders, fractures or fissures), previous neck surgeries, cervical radiculopathy or myelopathy and any other medical condition (e.g., fibromyalgia or tumour).

### 2.3. Asessments

Sociodemographic data (e.g., sex, age, height, weight and body mass index) was collected by using a standardized history. In addition, prior to the subjects’ participation in the study, neck pain intensity and neck disability were assessed. A 100 mm VAS was used for assessing the neck pain intensity. In this scale, “0” means complete absence of pain and “100” the worst imaginable pain. Cut-off scores were interpreted as follows: VAS < 34: mild pain, 35 < VAS < 64: moderate and 65 < VAS: severe [17]. Since the Neck Disability Index (NDI) is a valid tool for measuring perceived disability associated with neck pain [18], it was used for assessing the neck disability. This self-reported questionnaire consists of 10 items with a final score ranging 0-to-50 and expressed as percentage. Scores ranging 0–8% were considered as no disability, 10–28% as mild disability, 30–48% as moderate, 50–68% as severe and 70–100% as complete disability [19].

#### 2.3.1. Blinding

To ensure the methodological quality, three different blinded assessors were involved in this study: (1) The first assessor located and marked location of the points (control point, active MTrP and latent MTrP); (2) The second assessor measured the PPTs blinded to the participants’ group (symptomatic/asymptomatic) and point category (control point, active MTrP or latent MTrP); (3) The third assessor performed the SWE assessment blinded to the participants’ group, point category and PPT score.

#### 2.3.2. Myofascial Trigger Points Identification

**Figure 1:**
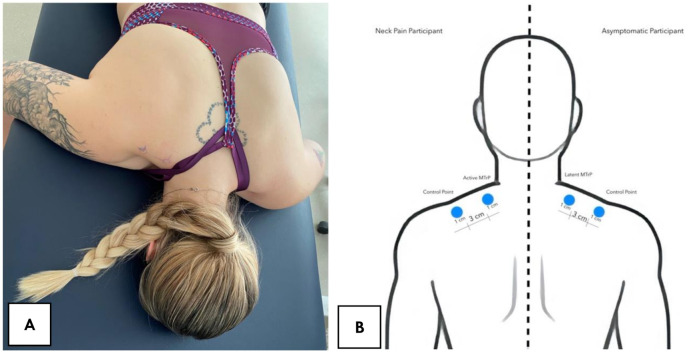
(**A**) Participants positioning; (**B**) Measurement points location.

Since manual palpation if performed by an experienced clinician is considered to reliably identify MTrP locations in the upper trapezius muscle by using a palpation protocol [20], a single experienced clinician (more than 10 years of experience in the assessment and treatment of MPS) conducted a physical examination to confirm the active or latent MTrP location by using a manual palpation protocol and assessment of the responses of the patients about specific questions about painful symptoms following the criteria for identifying active and latent MTrPs provided by Fernández-de-las-Peñas and Dommerholt [4]:

Participants were placed in the prone position and the examiner assessed bilaterally the entire length of the upper trapezius muscle by using a pincer palpation to locate (1) a palpable taut band, (2) a hypersensitive tender spot in the taut band and (3) a local twitch response provoked by the snapping palpation of the taut band. MTrPs that upon stimulation reproduced partially or totally familiar symptoms experienced by the patients were considered active MTrPs and those that upon stimulation did not reproduce any symptom experienced and the subject did not recognize the elicited symptom as familiar was considered as a latent MTrP [4]. If there was more than one active MTrP, the most symptomatic site was selected and marked with a blue skin marking pen. Then, a control point located 3 cm laterally to the MTrP following the fibers direction (within the taut band) was also marked with the same blue skin marking pen (Figure 1).

We selected this control point location based on the pain difference during the manual palpation and based on a previous study [21] reporting that there are electromyographic differences between MTrPs and control points separated 1cm following the fibers direction (within the same taut band) and located in the upper trapezius muscle (200 to 700 μV and 10 μV respectively). Examiners were blinded to this assignation of MTrP and control locations during the measurement procedures.

#### 2.3.3. Shear Wave Elastography-Image Acquisition

All ultrasound assessments were performed by the same examiner with more than 10 years of experience in musculoskeletal evaluation. A Canon Aplio A with a PLT-1005-BT 14L5 (5–14 MHz) transducer (Canon Medical Corp, 1385 Shimoishigami, Otawara, Tochigi 324-8550, Japan) were used for all the images capturing and measuring procedures. All participants were positioned in the same orientation used for the MTrP identification protocol. Since the longitudinal plane showed higher intra- and inter-examiner reliability (ICC = 0.66–0.74) than the transversal assessment [22], all images were acquired with the transducer oriented longitudinally with the muscle fibers and positioning the center of the probe over the MTrP and the control point locations. Two images were obtained from each patient and measurement point to improve the accuracy of the measurements.

**Figure 2:**
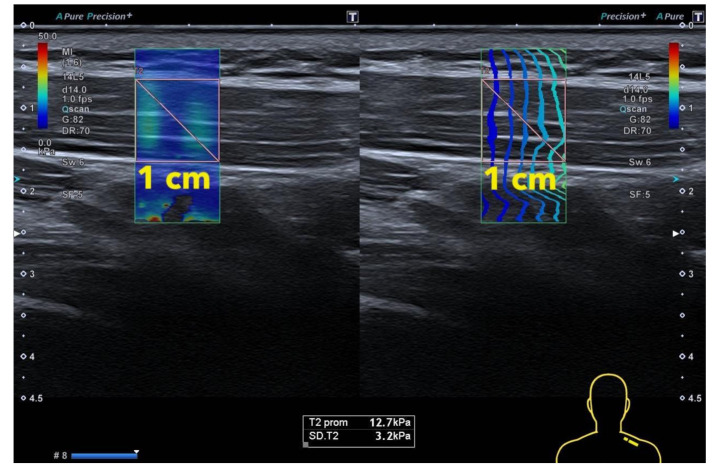
Shear Wave Elastography: Image measurement.

In order to avoid bias regarding the MTrP surface selection based on the stiffness scale, regions of interest (ROIs) for shear wave data were positioned to cover (1) the superficial and deep internal echogenic fascia of upper trapezius (in order to include completely the muscle thickness) and (2) 1 cm of width (correspondent with the algometer surface) (Figure 2). Finally, both the Young Modulus and the Shear Wave Speed data were collected. The sonographer was blinded to the sample group (symptomatic or asymptomatic) and the point assessed (MTrP or control).

#### 2.3.4. Pain Pressure Thresholds

Since algometry is a valid and reliable method for assessing pain sensitivity in the upper trapezius muscle [23], both the MTrP and control point PPTs were assessed by using a digital Wagner FDX algometer (with 1 cm^2^ surface area) increasing the pressure at a rate of 1 kg/s by a different single assessor blinded to the participants group (symptomatic or asymptomatic), PPT scores and point (MTrP or control). All participants received the same standardized instructions, “I am going to push on your body at 2 places. If you feel pain, not pressure, say ‘now’ and I will stop” [24]. The mean average of 3 trials was calculated for each point (MTrP and control; right and left sides) and analyzed.

#### 2.3.5. Pain Extent

**Figure 3:**
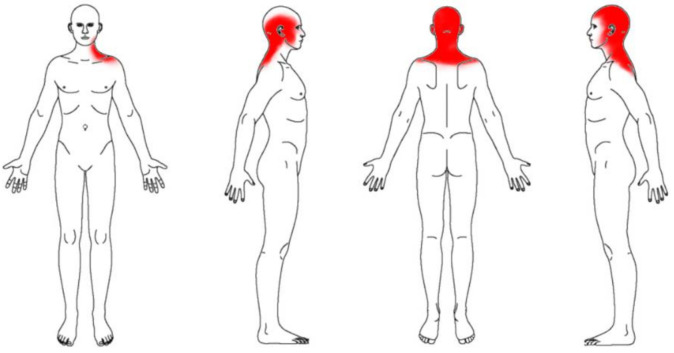
Body Chart used for pain extent.

For the calculation of the pain extent or pain area, a body chart was used since this procedure showed good reliability (ICC = 0.74) to assess pain sites. Participants were asked to paint with a red pen their pain area in 4 standard body chart images (anterior, both side lateral and posterior views). Participants were supervised by one researcher to avoid extra drawings. Then, all images were scanned with the same scanner, size and resolution to be analyzed in tiff format by using the ImageJ software [25]. Finally, their pain area was calculated as a percentage by dividing the red area by the total area of the 4 body charts (Figure 3).

### 2.4. Statistical Analysis

All statistical analyses were conducted in IBM SPSS Statistics V.25 for Mac OS (IBM Corporation, Armonk, NY, USA) setting at a significance level *p* < 0.05. Firstly, normal data distribution was verified by using the Shapiro-Wilk test. A descriptive analysis was conducted to describe the sociodemographic and clinical characteristics of the sample, by gender and by group (Symptomatic and Asymptomatic). Normal-distributed data were described by means and standard deviation (SD). For sociodemographic characteristics, between-groups differences by gender and group were assessed with Student t-test for independent samples. PPT and SWE side-to-side, MTrP-control and between-groups differences were also assessed by using Student t-tests. In order to assess the intra-examiner reliability of SWE and PPT measurements, a 2-way mixed-model, consistency-type intra class correlation coefficient (ICC_3,1_) was calculated. ICC scores were classified as fair (ICC < 0.50), moderate (0.50 < ICC < 0.75), good (0.75 < ICC < 0.90) or excellent (0.90 < ICC) [26].

Pearson’s correlation coefficient was used to calculate a multivariable correlation analysis between sociodemographic and clinical data. Pearson’s r scores were considered as “poor” if r < 0.3, “fair” if 0.3 < r< 0.6, “moderate” if 0.6 < r < 0.8 and strong if 0.8 < r [27]. In addition, r coefficients were used to identify multicollinearity and shared variance between the variables when r > 0.8.

## 3. Results

Sixty (*n* = 60) volunteers were initially recruited in April 2021. Five participants were excluded due to: being under pharmacological treatment (*n* = 3), previous neck surgery (*n* = 1) and radiculopathy diagnosis (*n* = 1). Fifty-three participants were finally included in this study. Thirty-eight active MTrPs, sixty-eight latent MTrPs and one hundred-six control points were analyzed. All neck pain patients (*n* = 19) showed bilateral active MTrP located in the upper trapezius. None of the participants were lost or excluded during the study. The intra-examiner reliability estimates were excellent for PPTs (ICC_3,1_ = 0.913–0.967) and good-to-excellent for SWE (ICC_3,1_ = 0.868–0.927).

Table 1 describes sociodemographic characteristics of the total sample, by gender and group. Although males presented significant BMI and height differences (*p* < 0.001) compared with females, no sociodemographic differences for neck pain and asymptomatic samples were found.

Table 2 shows the PPT of latent MTrPs and control points of the asymptomatic sample and the PPT of active MTrPs and control points, NDI, VAS and pain extent of the neck pain patients. For asymptomatic subjects, latent MTrPs and control locations showed no PPT differences (*p* > 0.05).

Neck pain patients showed significant PPT differences between the active MTrP and the control location (left side: *p* < 0.05; right side: *p* < 0.01; mean of both sides: *p* < 0.001). No side-to-side differences were found for asymptomatic subjects (latent MTrP and control point: *p* > 0.05) nor neck pain patients (active MTrP and control point: *p* > 0.05). In addition, between-groups differences for latent and active MTrPs were found (left side, right side and mean; *p* < 0.001). Finally, significant between-groups control point PPT differences were found (left: *p* < 0.05; right: *p* < 0.01; mean: *p* < 0.001).

Table 3 summarizes Young Modulus and Shear Wave Speed of active MTrPs (symptomatic sample), latent MTrPs (asymptomatic sample) and control locations (both samples). No stiffness differences (assessed as Young modulus nor shear wave speed) were found between latent MTrPs with control points (*p* > 0.05), active MTrPs with control points (*p* > 0.05) nor latent MTrPs with active MTrPs (*p* > 0.05). In addition, no side-to-side differences were found in the active MTrPs, latent MTrPs or control points stiffness (all, *p* > 0.05). However, significant between-groups differences were found for the Young modulus (right side: *p* < 0.05) and shear wave speed (right side and mean of both sides: *p* < 0.05).

Data regarding the correlation analysis between sociodemographic, clinical and SWE features are reported on Table 4. Young modulus showed no significant association with the sociodemographic and clinical outcomes assessed (all, *p* > 0.05). Although shear wave speed showed no association with clinical severity indicators (all, *p* > 0.05), it showed to be negatively correlated with weight and BMI (*p* < 0.05 and *p* < 0.01 respectively). Gender was associated with VAS (*p* < 0.01), PPT (*p* < 0.01), NDI (*p* < 0.01) and pain extent (*p* < 0.05); height was associated with VAS (*p* < 0.01), PPT (*p* < 0.01), NDI (*p* < 0.05) and pain extent (*p* < 0.05); weight was associated with VAS (*p* < 0.01), PPT (*p* < 0.01), and pain extent (*p* < 0.01) but not with NDI (*p* > 0.05); BMI was associated with VAS (*p* < 0.05), PPT (*p* < 0.01), and pain extent (*p* < 0.01) but not with NDI (*p* > 0.05) and age was not associated with the clinical severity indicators assessed (all, *p* > 0.05).

## 4. Discussion

### 4.1. Findings

This is the first study analyzing the clinical relevance of SWE as indicator of MPS severity. In this study, (1) we compared the stiffness of neck pain patients with active MTrPs with asymptomatic subjects with latent MTrPs, (2) we compared the stiffness of both latent and active MTrPs with control points and (3) we conducted a correlation analysis between stiffness assessed as Young modulus and shear wave speed and clinical severity assessed as pain intensity, pain sensitivity, disability and pain extent. The most interesting finding of this study was that, although there were important sensitivity differences between active MTrPs with latent MTrPs and control points, we found no stiffness differences. However, neck pain patients and asymptomatic subjects showed significant stiffness differences for the control point.

Since MTrP are classically defined as hard palpable nodules within confirmed taut bands [28] and are characterized by tenderness associated with pain [4], it would be logical find stiffness and PPT differences between active MTrPs, latent MTrPs and control points within the same muscle. However, even if MTrP showed an increased pain sensitivity (lower PPT) compared with control point locations, we did not find stiffness differences between active or latent MTrPs with control points. Our results are consistent with a previous study since it was reported that the trapezius muscle hardness does not directly reflect the subjective shoulder stiffness [29].

Since palpation is shown to be successful for identifying the most sensitive areas (active and latent MTrPs), is the most popular diagnosis procedure used for assessing stiffness differences. However, manual palpation is a high subjectivity test based on the pain response of the patients which could imply bias and, therefore, objective methods (with high sensitivity, specificity, reliability and validity) are prioritized [30]. Although SWE is an objective alternative to manual palpation which showed to be a feasible tool for assessing the upper trapezius muscle intra-session stiffness [22], the main limitation found for this method is that its reliability showed to be moderate just if performed in rigorous conditions (e.g., reporting both shear wave speed and young modulus assessed in the longitudinal plane [31]. Therefore, those studies assessing transverse planes [32,33] or using operator-dependent elastography methods (e.g., strain elastography) should be interpreted carefully [11].

A previous study conducted by Ballyns et al. [11] reported no correlation between trigger point areas and PPTs for either latent or active MTrPs. Therefore, even if they found that active MTrPs show greater size compared with latent MTrPs, there are other mechanisms contributing to the trigger point sensitivity such as sensitizing biochemicals as reported by Shah et al. [34] derived from the capillary constriction consistent with Doppler US findings [11]. Our results are consistent with this hypothesis since we found in addition to the sensitivity differences between healthy and clinical populations regarding the control point locations, greater stiffness scores in neck pain patients.

We also found stiffness differences in the control point location between neck pain patients and asymptomatic subjects. This is an interesting finding since the general muscle stiffness showed to be a more differential factor compared with the MTrP stiffness. Future studies could include the general muscle stiffness as an additional outcome to assess the effects of different interventions on the general stiffness and its correlation with other clinical features. In fact, emerging evidence is considering this proposal and considering the SWE as a tool for clinical evaluation and monitorization of treatment responses [35].

Although our results suggest that SWE cannot be used as clinical severity indicator or a tool to differentiate active and latent MTrPs based on Young modulus and shear wave speed, this field requires further investigation to consolidate these findings. In fact, we found no correlation between age and stiffness. One likely reason explaining the discrepancy with the current evidence [36] is the small range of ages included in this study. Therefore, our results cannot be extrapolated to the general population and further research should consider greater ranges of age, height, weight, BMI and pain intensity/disability to confirm these findings.

### 4.2. Limitations

Even if the results of this study are promising and we considered the blinding of the assessors and the inclusion of one sample of asymptomatic subjects as a control group, some potential limitations should be recognized. Firstly, we measured the locations stiffness just in the longitudinal plane due to reliability restrictions previously reported. Since the image assessed limited fibers and MTrP are located in taut bands, this could show increased stiffness in the control points selected. Secondly, additional associations between SWE with Doppler US, electromyography and biochemical analysis would clarify several aspects of our findings and confirm the presence of active or latent MTrPs. Finally, the sample was relatively small and further research including greater samples with wide standard deviations is needed.

## 5. Conclusions

This study showed that active MTrPs located in the upper trapezius identified by manual palpation in neck pain patients are more sensitive compared with control point within the same muscle and with latent MTrPs in asymptomatic subjects. We found no stiffness differences assessed as SWE Young modulus and shear wave speed between active and latent MTrPs. However, neck pain patients showed increased control point stiffness compared with asymptomatic subjects. Therefore, future studies could assess how different interventions change the general muscle stiffness (not focusing just in MTrPs) and its relevance during the clinical evaluation and follow-up by analyzing the correlation between clinical features with the general stiffness (control points), specific stiffness (MTrPs) and stiffness change. Finally, we found no association between SWE features with clinical severity indicators of MPS assessed as pain extent, pain intensity, sensitivity and neck disability.

## Figures and Tables

**Table 1:** Sociodemographic characteristics of the total sample, by gender, and group.

	Subjects (*n*)	Age (Years)	Height(m)	Weight(kg)	BMI (kg/m^2^)
Total sample	53	21.7 ± 4.9	1.71 ± 0.09	67.3 ± 13.1	22.6 ± 2.9
Gender					
Males	21	23.1 ± 2.8	1.79 ± 0.09 *	77.9 ± 11.9 *	24.0 ± 2.8 *
Females	32	20.8 ± 5.7	1.66 ± 0.09	60.5 ± 8.7	21.7 ± 2.6
Group					
Neck Pain	19	21.5 ± 3.2	1.74 ± 0.07	74.1 ± 13.1	23.6 ± 3.0
Asymptomatic	34	21.9 ± 5.9	1.70 ± 0.10	64.0 ± 12.3	21.9 ± 2.6

* Significant differences between groups (*p* < 0.001).

**Table 2:** Clinical characteristics of the participants.

Asymptomatic Subjects						
	PPT (kg/cm^2^)			NDI (%)	VAS (0–100)	Pain Extent (%)
	MTrP ^a^	Control	Difference			
Mean	3.13 ± 1.09	3.55 ± 0.95	0.42 (−0.04; 0.89)	-	-	-
Left	3.12 ± 1.19	3.37 ± 0.71	0.25 (−0.39; 0.90)			
Right	3.14 ± 1.02	3.73 ± 1.13	0.59 (−0.11; 1.30)			
*Between sides difference*	0.02 (−0.70; 0.75)	0.36 (−0.26; 0.98)				
Neck Pain Subjects						
Mean	1.84 ± 0.85	2.57 ± 1.40	0.73 (0.31;1.14) *	17.08± 7.49	41.8 ± 11.0	2.67 ± 1.60
Left	1.82 ± 0.88	2.50 ± 1.45	0.68 (0.05; 1.30) ‡			
Right	1.86 ± 0.84	2.64 ± 1.37	0.78 (0.20; 1.36) †			
*Between sides difference*	0.04 (−0.40; 0.48)	0.14 (−0.58; 0.86)				
Between-Groups Differences						
Mean	1.29 (0.90; 1.68) *	0.98 (0.46; 1.49) *				
Left	1.30 (0.70; 1.89) *	0.87 (0.15; 1.59) ‡				
Right	1.28 (0.74; 1.81) *	1.09 (0.33; 1.85) †				

* *p* < 0.001; † *p* < 0.01; ‡ *p* < 0.05, ^a^ Latent MTrP for asymptomatic subjects and Active MTrP for neck pain subjects.

**Table 3:** Shear Wave Elastography Characteristics.

Asymptomatic Subjects						
	Young Modulus (kPa)			Shear Wave Speed (m/s)		
	MTrP ^a^	Control Point	Difference	MTrP ^a^	Control Point	Difference
Mean	14.77 ± 5.25	13.86 ± 3.00	0.91 (−1.04; 2.86)	2.06 ± 0.43	2.02 ± 0.29	0.03 (−0.12; 0.20)
Left	14.43 ± 6.23	14.01 ± 3.36	0.41 (−2.88; 3.71)	2.00 ± 0.50	2.01 ± 0.35	0.01 (−0.28; 0.29)
Right	15.11 ± 4.18	13.71 ± 2.68	1.40 (−0.91; 3.72)	2.12 ± 0.35	2.03 ± 0.22	0.08 (−0.10; 0.28)
*Between sides difference*	0.68 (−2.81; 4.17)	0.30 (−2.30; 1.69)		0.12 (−0.16; 0.40)	0.02 (−0.16; 0.22)	
Neck Pain Subjects						
Mean	14.35 ± 4.22	15.48 ± 5.24	1.13 (−0.57; 2.83)	2.09 ± 0.32	2.17 ± 0.36	0.08 (−0.04; 0.20)
Left	13.61 ± 3.91	14.48 ± 4.14	0.86 (−1.21; 2.95)	2.02 ± 0.30	2.10 ± 0.30	0.07 (−0.08; 0.22)
Right	15.06 ± 4.44	16.45 ± 6.03	1.38 (−1.30; 4.07)	2.12 ± 0.34	2.24 ± 0.40	0.09 (−0.09; 0.28)
*Between sides difference*	1.45 (−0.69; 3.60)	1.96 (−0.69; 4.62)		0.12 (−0.04; 0.28)	0.14 (−0.04; 0.32)	
Between-Groups Differences						
Mean	0.42 (−1.48; 2.32)	1.62 (−0.23; 3.47)		0.02 (−0.12; 0.17)	0.14 (0.00; 0.28) *	
Left	0.81 (−2.09; 3.72)	0.46 (−1.80; 2.74)		0.02 (−0.20; 0.25)	0.08 (−0.10; 0.27)	
Right	0.04 (−2.50; 2.59)	2.74 (−0.21; 5.69) *		0.02 (−0.18; 0.22)	0.20 (0.00; 0.40) *	

* *p* < 0.05, ^a^ Latent MTrP for asymptomatic subjects and Active MTrP for neck pain subjects.

**Table 4:** Pearson-product moment correlation matrix.

	1	2	3	4	5	6	7	8	9	10
1. Gender										
2. Height	−0.659 **									
3. Weight	−0.644 **	0.752 **								
4. BMI	−0.381 **	0.260 **	0.826 **							
5. Age	−0.231 **	0.174 *	n.s.	n.s.						
6. Young Modulus	n.s.	n.s.	n.s.	n.s.	n.s.					
7. Shear Wave Speed	n.s.	n.s.	−0.141 *	−0.230 **	n.s.	0.937 **				
8. VAS	0.324 **	−0.194 **	−0.230 **	−0.175 *	n.s.	n.s.	n.s.			
9. NDI	0.200 **	−0.152 *	n.s.	n.s.	n.s.	n.s.	n.s.	0.859 **		
10. PPT	−0.386 **	0.193 **	0.258 **	0.185 **	n.s.	n.s.	n.s.	−0.417 **	−0.362 **	
11. Pain Extent	0.177 *	−0.195 *	−0.273 **	−0.301 **	n.s.	n.s.	n.s.	0.295 **	0.359 **	n.s.

n.s. non-significant, * *p* < 0.05; ** *p* < 0.01.

## Data Availability

The data that support the findings of this study are available from the corresponding author (JA Valera-Calero), upon reasonable request.
